# A Case of Pancreaticoduodenectomy for Grade V Traumatic Pancreatic Injury in an Elderly Patient

**DOI:** 10.7759/cureus.79533

**Published:** 2025-02-23

**Authors:** Tsuyoshi Terada, Susumu Matsushime, Keisuke Kamo, Kazuki Hashida, Nobuichiro Tamura

**Affiliations:** 1 Department of General Surgery, Kurashiki Central Hospital, Kurashiki, JPN; 2 Department of Emergency Medicine, Kurashiki Central Hospital, Kurashiki, JPN; 3 Department of Surgery, Institute of Gastroenterology, Tokyo Women's Medical University, Shinjuku, JPN

**Keywords:** damage control surgery, grade v, pancreaticoduodenectomy(pd), traumatic pancreatic injury, whipple's procedure

## Abstract

Traumatic pancreatic injury is a rare condition, but cases involving main pancreatic duct injury often require surgical intervention and are associated with high mortality rates. Recently, two-stage surgical approaches, with initial damage control surgery followed by delayed pancreatic resection and reconstruction, have been increasingly reported. However, we argue that not all cases need a two-stage approach; instead, surgical strategies should be tailored based on the patient's vital signs. Furthermore, in pancreatic surgery, effective collaboration between trauma surgeons and hepatopancreatobiliary (HPB) surgeons is essential. Here, we report a case of a grade V traumatic pancreatic injury in an elderly patient that was successfully managed with one-stage pancreaticoduodenectomy, performed through coordinated efforts between trauma and HPB surgeons, resulting in a favorable outcome.

## Introduction

Traumatic injuries to the pancreas are relatively uncommon, accounting for approximately 5% of abdominal trauma cases [1]. In Japan, nearly 90% of these injuries are caused by blunt trauma [2]. The retroperitoneal location of the pancreas often leads to delayed diagnosis, resulting in a mortality rate of 13-50%, which increases to over 73% in cases involving pancreatic head injuries [1,3]. A critical factor influencing prognosis is the presence of a main pancreatic duct injury [4]. Pancreatic injuries involving the main pancreatic duct, classified as grades III, IV, and V according to the Organ Injury Scale of the American Association for the Surgery of Trauma (AAST-OIS), have traditionally required surgical intervention [5].

The prognosis of emergent pancreatic resections has been shown to be worse compared to elective procedures [6]. Recently, damage control surgery (DCS) has become a more common initial approach, followed by staged pancreatic resection and reconstruction [7]. DCS is the preferred treatment for traumatic pancreatic injury with hemodynamic instability, as it enables rapid stabilization and early acid-base correction [1]. Thompson et al. suggest that staged pancreaticoduodenectomy (PD) after DCS benefits from pancreatic hardening, facilitating reconstruction [1]. In recent years, some trauma surgeons have recommended that all traumatic pancreatic injuries be managed with a staged approach [8]. However, Thompson et al. found no significant difference in complication rates between staged and primary PD [1]. Therefore, primary PD remains a feasible option for hemodynamically stable patients.

In this report, we present a case of a grade V traumatic pancreatic injury in an elderly patient who successfully underwent a single-stage PD with the involvement of both trauma and hepatopancreatobiliary (HPB) teams. In recent years, DCS has become widely adopted, and trauma surgeons now occasionally manage liver and pancreatic injuries without the involvement of HPB surgeons [8]. However, this case demonstrates that collaboration between trauma and HPB surgeons remains essential in the management of traumatic pancreatic injury, even in the era of DCS.

## Case presentation

An 85-year-old woman with hypertension and dyslipidemia was admitted to the emergency department 50 minutes after crashing her vehicle into an object. She was wearing a seatbelt, and the airbags deployed. On arrival, her vital signs were as follows: blood pressure, 108/45 mmHg; heart rate, 127 bpm; respiratory rate, 36 breaths/min; and temperature, 36.3°C. Her Glasgow Coma Scale score was 14. Abdominal examination showed no visible contusions or lacerations. A focused assessment with sonography for trauma detected fluid collections in Morrison's and Douglas' pouches. After 500 mL of intravenous fluids, her blood pressure improved to 143/94 mmHg, and her heart rate decreased to 59 bpm. Her hemodynamics stabilized without the need for blood transfusions or vasopressor support, but a blood transfusion was prepared just in case.

Blood biochemistry tests showed elevated liver and pancreatic enzymes (aspartate aminotransferase (AST), 205 U/L; alanine aminotransferase (ALT), 150 U/L; amylase (AMY), 178 U/L; and lipase, 365 U/L) (Table 1).

**Table 1:** Blood test results on arrival at the emergency department Blood biochemistry tests revealed elevated liver and pancreatic enzyme levels. IFCC: International Federation of Clinical Chemistry

Parameter	Value	Normal range
Hemoglobin (g/dL)	10.7	11.3-15.2
Hematocrit (%)	33.5	33.4-44.9
Platelet count (×10^4^/μL)	20.2	13.0-36.9
Leukocyte count (/μL)	6800	3500-9100
Neutrophil percentage (%)	57.8	40-70
Serum glucose (mg/dL)	259	70-109
Blood urea nitrogen (mg/dL)	21	8-22
Serum creatinine (mg/dL)	0.61	0.47-0.79
Serum sodium (mmol/L)	139	136-147
Serum potassium (mmol/L)	4.6	3.6-5.0
Serum chloride (mmol/L)	103	98-109
Total bilirubin (mg/dL)	0.4	0.3-1.2
Alanine transaminase (U/L)	205	10-40
Aspartate transaminase (U/L)	150	5-40
Lactate dehydrogenase (U/L)	550	124-222
Alkaline phosphatase (IFCC) (U/L)	71	38-113
Gamma-glutamyl transferase (U/L)	24	≦30
Amylase (U/L)	178	37-125
Lipase (U/L)	365	13-55
Creatine kinase (U/L)	148	45-163
Activated partial thromboplastin time (sec)	25	24.3-36.0
Prothrombin time (sec)	13.1	10.5-13.5
Prothrombin time-international normalized ratio	1	0.85-1.15
Fibrinogen (mg/dL)	268	150-400

**Figure 1:**
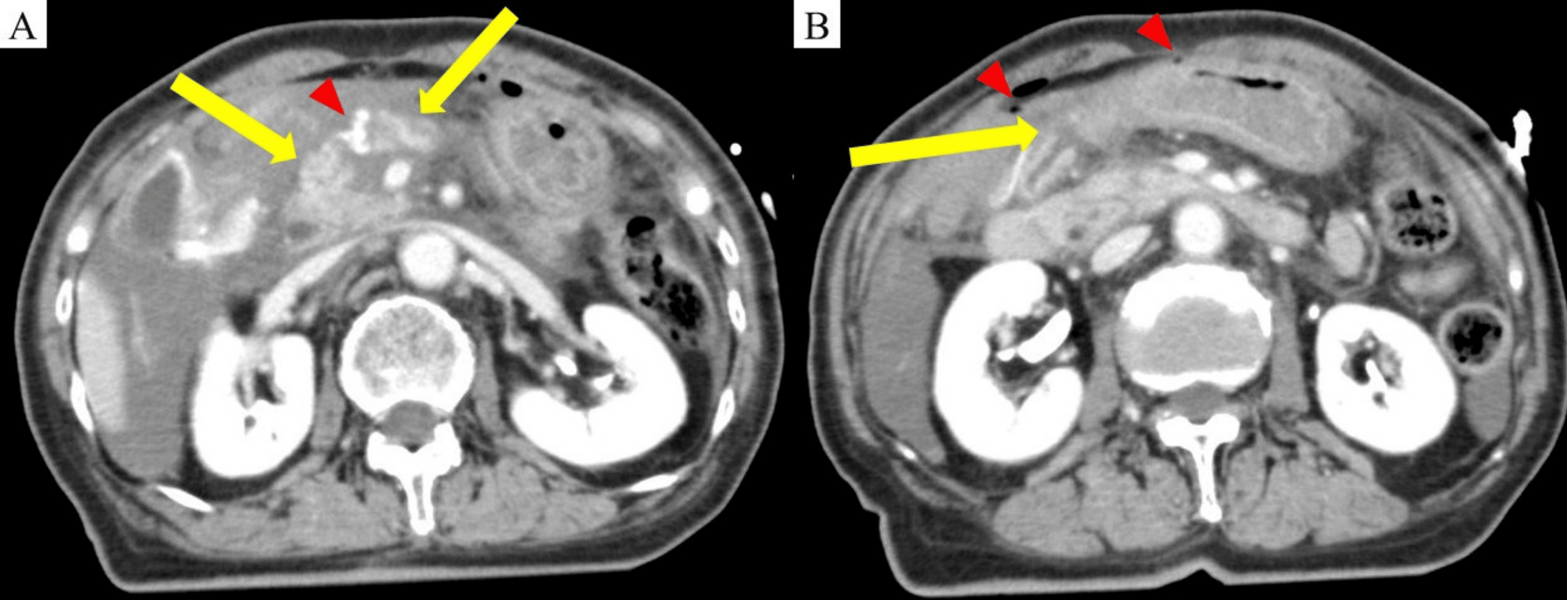
Contrast-enhanced abdominal computed tomography in the portal venous phase (coronal section) (A) A complete transection of the pancreas is observed just above the superior mesenteric vein (yellow arrow). Active bleeding from the distal right gastric artery was inferred (red triangle). (B) Disruption of mucosal continuity in the duodenal bulb is noted (yellow arrow). Additionally, free air is present in the abdominal cavity (red triangle).

Contrast-enhanced abdominal computed tomography revealed a complete transection of the pancreatic body at the portal vein level (Figure 1A), duodenal bulb mucosal discontinuity, and intra-abdominal free air (Figure 1B).

Active bleeding from the distal right gastric artery and fluid accumulation from the hepatic hilum to Morrison's pouch were inferred. Additional findings included traumatic subarachnoid hemorrhage, a left distal ulnar fracture, and abrasions at the left oral commissure and wrist.

The patient was diagnosed with a grade V pancreatic injury, a grade II liver injury, and a right gastric artery injury according to the AAST-OIS classification. An exploratory laparotomy was planned with collaboration between the trauma and HPB teams.

**Figure 2:**
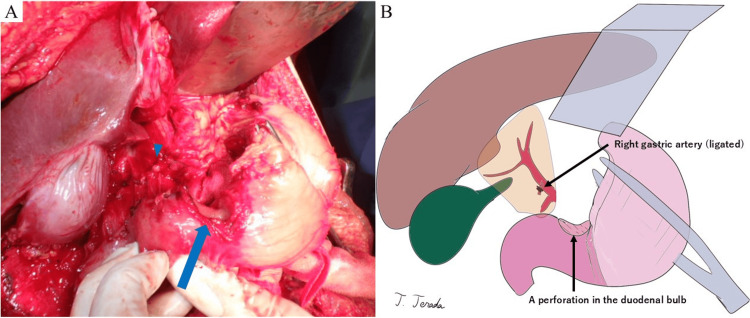
Intraoperative photograph and corresponding illustration (A) A perforation in the duodenal bulb is observed (blue arrow). Active bleeding from the right gastric artery is noted and successfully controlled by ligation and division (blue triangle). (B) An illustration created by the author using the Procreate® app (Savage Interactive, Hobart, Australia) on an iPad® (Apple Inc., Cupertino, CA, USA).

After the administration of 1,200 mL of intravenous fluids, laparotomy was initiated 82 minutes after arrival. Tracheal intubation was performed immediately before surgery, and a red blood cell (RBC) transfusion was initiated. During the laparotomy, a grade II liver laceration was managed with gauze packing, and the right gastric artery was ligated to stop the bleeding (Figure 2). The perforation site in the duodenal bulb was identified (Figure 2). The duodenal perforation was extensive, and complete closure was deemed to require the resection of the contused area. However, to prevent contamination at least in the short term from pancreatic fluid and bile, an initial closure was performed using interrupted sutures with 3-0 monofilament.

**Figure 3:**
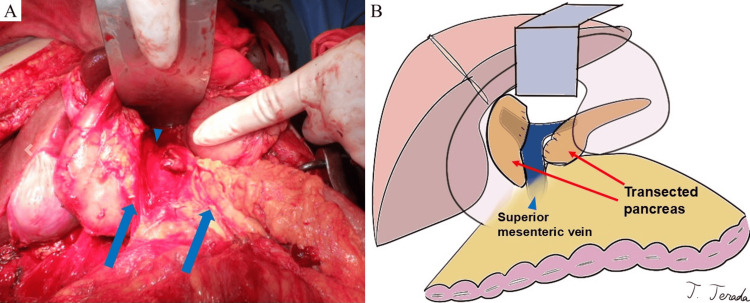
Intraoperative photograph and corresponding illustration (A) A complete transection of the pancreas (blue arrow) is observed just above the superior mesenteric vein (blue triangle). (B) An illustration created by the author using the Procreate® app (Savage Interactive, Hobart, Australia) on an iPad® (Apple Inc., Cupertino, CA, USA).

The omental bursa was then opened, and the Cattell-Braasch maneuver was performed, revealing a complete transection of the pancreas just above the superior mesenteric vein (Figure 3). Severe disruption made it difficult to identify the main pancreatic duct. No additional vascular or intestinal injuries were found.

**Figure 4:**
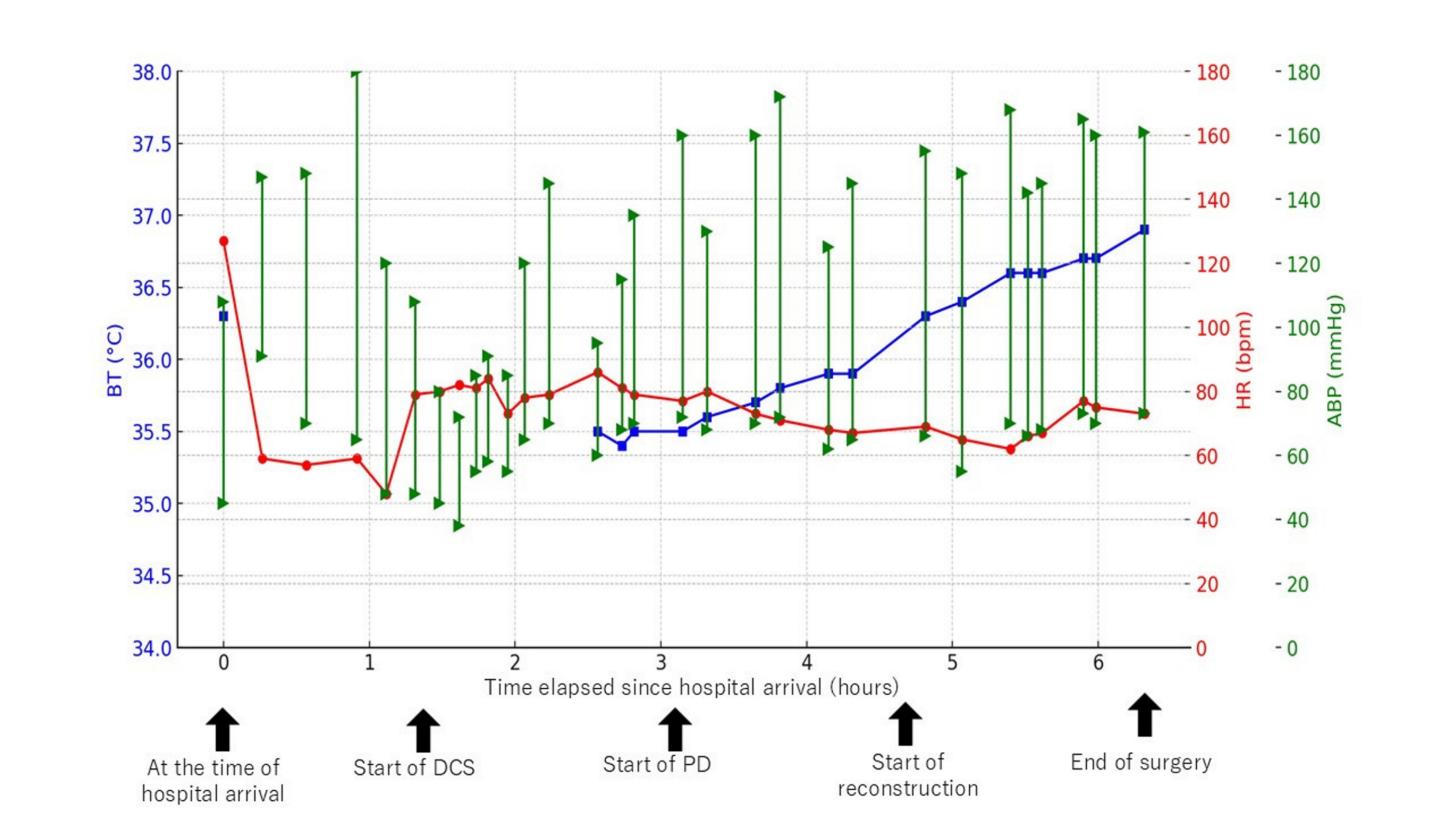
Trend of vital signs after hospital arrival This figure illustrates the trend of vital signs following hospital arrival. The x-axis represents the elapsed time from hospital arrival (hours), and the y-axis represents the respective values of each vital sign. Blue squares represent body temperature. Red circles represent heart rate. Green triangles indicate both systolic and diastolic arterial blood pressure. BT: body temperature; HR: heart rate; ABP: arterial blood pressure; DCS: damage control surgery; PD: pancreaticoduodenectomy

The initial stabilization phase, referred to as DCS, was led by the trauma surgery team and lasted one hour and 37 minutes. During this period, the patient's systolic blood pressure remained above 100 mmHg without vasopressor support, and core temperature was maintained above 35°C. She received eight units (1120 mL) of RBCs, eight units (960 mL) of fresh frozen plasma (FFP), and 10 units (200 mL) of platelets. At the completion of DCS, the patient remained normothermic (35.7°C), with no acidosis (pH 7.358) or coagulopathy (Figure 4).

The trauma team initially planned to conclude the surgery as DCS. However, given the difficulty in identifying the main pancreatic duct, the inevitability of pancreatic fistula, and concerns about bile leakage due to duodenal perforation, the HPB team was consulted for definitive surgical management.

Considering the severe crush injury to the pancreatic head and body and the extent of duodenal damage, the decision was made to perform a subtotal stomach-preserving PD with additional pancreatic body resection to facilitate main pancreatic duct identification. An additional two units (280 mL) of RBCs were administered intraoperatively. The patient remained hemodynamically stable throughout the procedure, with no hypothermia (36.6°C), acidosis (pH 7.410), or coagulopathy, confirming the feasibility of a one-stage reconstruction (Figure 4, Table 2, Table 3).

**Table 2:** Trend of arterial blood gas data after hospital arrival This table illustrates the changes in arterial blood gas data over time after hospital arrival. pO2: partial pressure of oxygen; pCO2: partial pressure of carbon dioxide; HCO3-: bicarbonate; DCS: damage control surgery; PD: pancreaticoduodenectomy

	Initial arterial blood gas	Start of DCS	Start of PD	Start of reconstruction	End of surgery
Elapsed time since hospital arrival	0.42 hours	1.37 hours	3.05 hours	4.60 hours	6.48 hours
pH	7.409	7.408	7.358	7.410	7.445
pO2 (mmHg)	245	485	212	218	210
pCO2 (mmHg)	39.6	39	42.8	39.3	37.9
HCO3- (mmol/L)	24.6	24.1	23.5	24.5	25.7
Base excess (mmol/L)	0.5	0.1	-1.4	0.4	2.1
Lactate (mmol/L)	1.6	1.8	3.2	3.4	2.9

**Table 3:** Perioperative laboratory parameters This table illustrates the changes in blood test parameters during surgery.

	Start of pancreatoduodenectomy	Start of reconstruction	End of surgery
Hemoglobin (g/dL)	11.2	8.2	10.8
White blood cell count (×10³/μL)	11.1	6.9	7.6
Platelet count (×10⁴/μL)	10.1	12	9.1
Activated partial thromboplastin time (sec)	27.1	30.2	28.1
Prothrombin time (sec)	15.1	15.2	14.2
Prothrombin time-international normalized ratio	1.12	1.15	1.07
Fibrinogen (mg/dL)	170	175	197

**Figure 5:**
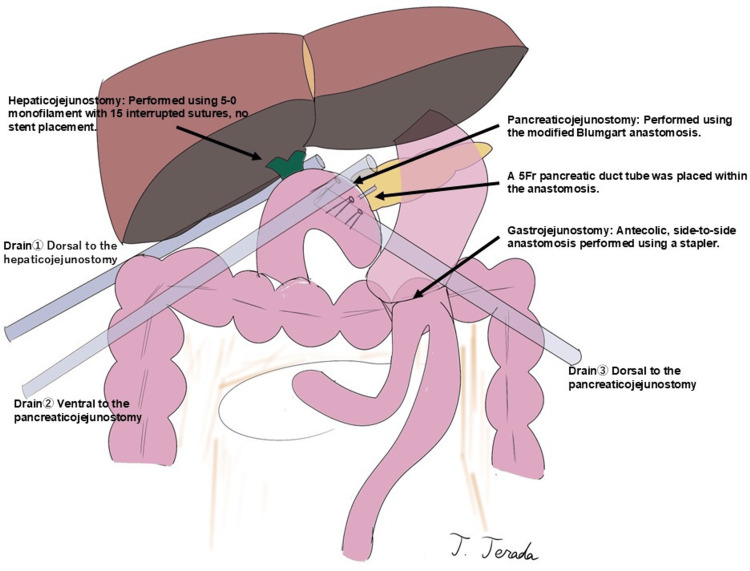
Reconstruction following pancreaticoduodenectomy using the modified Child method Schematic representation of the reconstruction performed following pancreaticoduodenectomy using the modified Child method. A pancreaticojejunostomy was created using the modified Blumgart method, with a 5Fr pancreatic duct stent placed intraluminally. Hepaticojejunostomy was performed with interrupted suturing using 5-0 monofilament, and gastrojejunostomy was completed using a surgical stapler. No external fistula or enterostomy tubes were placed. Three intra-abdominal drains were inserted before abdominal closure to ensure adequate postoperative drainage.

Reconstruction was performed using the modified Child method. A pancreaticojejunostomy was created using the modified Blumgart method, with a 5Fr pancreatic duct stent placed intraluminally. Hepaticojejunostomy was performed using an interrupted suturing with 5-0 monofilament, and gastrojejunostomy was completed using a surgical stapler. No external fistula or enterostomy tubes were placed. Three intra-abdominal drains were inserted before abdominal closure, completing the procedure (Figure 5).

The total operative time was five hours and seven minutes, with an estimated blood loss of 2,835 mL. Intraoperatively, the patient required 14 units (1960 mL) of packed RBCs, 10 units (1200 mL) of FFP, and 10 units (200 mL) of concentrated platelets (Figure 4, Table 2).

The patient's postoperative course was uneventful. She was extubated on postoperative day 2 and began oral intake on postoperative day 6. No pancreatic or biliary fistulas were observed. Although she regained her preoperative level of activities of daily living (ADL) and was functionally independent, she required social support due to living alone. Following surgical fixation of a left ulnar fracture, she was discharged to a rehabilitation facility on postoperative day 41.

At the two-year follow-up, the patient, now 87 years old, remains independent in ADLs. However, she has experienced three episodes of cholangitis due to a biliary-enteric anastomotic stricture, which has been managed conservatively. Her diabetes has slightly worsened but remains controlled with oral hypoglycemic agents, and her exocrine function is preserved with oral pancrelipase supplementation.

## Discussion

This case demonstrates that single-stage PD for severe traumatic pancreatic injury with complete pancreatic transection and duodenal perforation (AAST-OIS grade V) remains feasible, even in the era where DCS is widely adopted, provided that the patient is hemodynamically stable and the procedure is performed by surgeons experienced in pancreatic surgery.

Traumatic pancreatic injuries involving the main pancreatic duct (AAST-OIS grades III-V) traditionally require surgical intervention; however, their prognosis remains poor, with mortality rates ranging from 13% to 50% [1,4]. To overcome these challenges, DCS and staged repair of injury have been proposed and implemented [1,8].

DCS is a surgical strategy designed to avoid high-risk procedures, such as complex reconstructions or anastomoses, in trauma patients presenting in shock. Instead, organ repair and reconstruction are deferred to a second stage [9]. The most widely accepted criteria for performing DCS are the so-called "lethal triad," which consists of hypothermia (median temperature <34°C), acidosis (median pH <7.2), and coagulopathy [10].

In the management of traumatic pancreatic injury, the first consideration for both trauma surgeons and HPB surgeons should be the patient's survival, and for this reason, DCS and staged repair of injury should always be considered [1]. At our institution, two-stage PD is performed not only in emergency surgeries for traumatic pancreatic injuries but also in elective surgeries for pancreatic cancer. In fact, we have previously reported a case in which we employed open abdominal management for staged reconstruction following massive portal vein bleeding during PD with portal vein resection for pancreatic head cancer [11].

However, it is essential to recognize that DCS can lead to intra-abdominal abscesses, enterocutaneous fistulas, ventral hernias, and prolonged ICU stays [12]. Furthermore, inadequate drainage during DCS increases the risk of complications and must be carefully managed [2,13]. Thompson et al. suggest that during the second stage of surgery for pancreatic injury, the pancreas becomes hard due to pancreatitis that develops during the drainage period [1]. While PD for a hard pancreas is associated with a lower risk of postoperative pancreatic fistula, it has been reported to increase the likelihood of exocrine pancreatic insufficiency [14]. The primary objective of trauma surgery is survival; however, traumatic pancreatic injury typically occurs in younger patients compared to pancreatic cancer [15]. Considering long-term patient quality of life (QOL), performing reconstructive surgery while the pancreas remains soft may help reduce the risk of exocrine pancreatic insufficiency.

For these reasons, PD for grade V traumatic pancreatic injury with duodenal perforation remains feasible if the following conditions are met: hemodynamic stability of the patient, the presence of HPB surgeons experienced in PD, and a facility equipped to effectively manage postoperative complications of PD [16].

PD has been shown to achieve superior outcomes when performed at high-volume centers specializing in HPB surgery [16]. Similarly, trauma surgeries conducted in dedicated abdominal emergency centers have been associated with improved prognoses [2]. The management of traumatic pancreatic injuries, which require both advanced systemic trauma care and highly specialized surgical expertise, underscores the critical importance of close collaboration between HPB surgeons and trauma surgeons to optimize patient outcomes [7,17].

In this case, late biliary stenosis at the choledochojejunostomy site resulted in recurrent episodes of cholangitis. Non-dilated bile ducts are recognized as a risk factor for benign biliary stenosis following PD [18], and similar outcomes have been reported in cases of traumatic pancreatic injuries involving non-dilated bile ducts [19]. PD for traumatic pancreatic injuries is more frequently performed in younger patients compared to PD for malignant tumors [15]. Consequently, the management and prevention of biliary stenosis remain significant challenges that require further investigation.

As the aging population continues to grow, the number of traumatic pancreatic injury cases in elderly patients, such as this case, is expected to increase. In these cases, treatment strategies should focus not only on survival but also on maintaining postoperative ADLs. The primary objective in the management of traumatic pancreatic injury is to ensure patient survival, with DCS being a fundamental consideration [1]. While some advocate that all cases of traumatic pancreatic injury should be managed with DCS and staged repair of injury [8], we believe that even in the era where DCS is widely adopted, single-stage PD may be a feasible option in select cases and beneficial in preserving ADLs and exocrine pancreatic function.

## Conclusions

The present case demonstrated that one-stage PD for grade V traumatic pancreatic injury in elderly patients can be safely performed when vital signs are stable, in collaboration with HPB surgeons. In the management of severe traumatic pancreatic injury, DCS and staged repair of injury should always be considered, and single-stage PD may not be the first-line treatment. However, as demonstrated in this case, single-stage PD may be a feasible option and beneficial in preserving ADLs under specific conditions.

## References

[1] Thompson CM, Shalhub S, DeBoard ZM, Maier RV (2013). Revisiting the pancreaticoduodenectomy for trauma: a single institution's experience. J Trauma Acute Care Surg.

[2] Ando Y, Okano K, Yasumatsu H (2021). Current status and management of pancreatic trauma with main pancreatic duct injury: a multicenter nationwide survey in Japan. J Hepatobiliary Pancreat Sci.

[3] Ho VP, Patel NJ, Bokhari F (2017). Management of adult pancreatic injuries: a practice management guideline from the Eastern Association for the Surgery of Trauma. J Trauma Acute Care Surg.

[4] Bradley EL 3rd, Young PR Jr, Chang MC (1998). Diagnosis and initial management of blunt pancreatic trauma: guidelines from a multiinstitutional review. Ann Surg.

[5] Moore EE, Cogbill TH, Malangoni MA (1990). Organ injury scaling, II: pancreas, duodenum, small bowel, colon, and rectum. J Trauma.

[6] Rozich NS, Morris KT, Garwe T (2019). Blame it on the injury: trauma is a risk factor for pancreatic fistula following distal pancreatectomy compared with elective resection. J Trauma Acute Care Surg.

[7] Jeong SY, Lee Y, Lee H (2023). Pancreaticoduodenectomy as an option for treating a hemodynamically unstable traumatic pancreatic head injury with a pelvic bone fracture in Korea: a case report. J Trauma Inj.

[8] Nagashima F, Inoue S, Matsui D, Bansyoutani Y, Tokuda R, Fuzisaki O, Kobayashi M (2021). Efficacy of damage control surgery and staged endoscopic pancreatic ductal double stenting therapy for severe pancreatic head injury: a case report. J Med Case Rep.

[9] Rotondo MF, Schwab CW, McGonigal MD (1993). 'Damage control': an approach for improved survival in exsanguinating penetrating abdominal injury. J Trauma.

[10] Roberts DJ, Bobrovitz N, Zygun DA (2016). Indications for use of damage control surgery in civilian trauma patients: a content analysis and expert appropriateness rating study. Ann Surg.

[11] Kamihata K, Kitagawa H, Muto J (2022). A case of two-stage reconstruction with a 12-hour interval for severe intestinal congestion due to portal clamping during pancreaticoduodenectomy. Jpn J Gastroenterol Surg.

[12] Lamb CM, MacGoey P, Navarro AP, Brooks AJ (2014). Damage control surgery in the era of damage control resuscitation. Br J Anaesth.

[13] Seamon MJ, Kim PK, Stawicki SP, Dabrowski GP, Goldberg AJ, Reilly PM, Schwab CW (2009). Pancreatic injury in damage control laparotomies: is pancreatic resection safe during the initial laparotomy?. Injury.

[14] Nakamura H, Murakami Y, Uemura K, Hayashidani Y, Sudo T, Ohge H, Sueda T (2009). Predictive factors for exocrine pancreatic insufficiency after pancreatoduodenectomy with pancreaticogastrostomy. J Gastrointest Surg.

[15] Hyser E, Sahhar HS, Woollen C (2019). Modified Whipple on an 18-month-old with traumatic pancreatic transection and duodenal rupture. Trauma Case Rep.

[16] Gouma DJ, van Geenen RC, van Gulik TM, de Haan RJ, de Wit LT, Busch OR, Obertop H (2000). Rates of complications and death after pancreaticoduodenectomy: risk factors and the impact of hospital volume. Ann Surg.

[17] Naragund AV, Muddasetty R, Kumar SS (2022). Revisiting the conundrum: a case report on trauma Whipple's pancreaticoduodenectomy. Cureus.

[18] Duconseil P, Turrini O, Ewald J, Berdah SV, Moutardier V, Delpero JR (2014). Biliary complications after pancreaticoduodenectomy: skinny bile ducts are surgeons' enemies. World J Surg.

[19] Israr S, Rubalcava NS, Weinberg JA, Jones M, Gillespie TL (2018). Management of biliary stricture following emergent pancreaticoduodenectomy for trauma: report of two cases. Cureus.

